# A commentary on encountering austerity; the experiences of four men living in a London hostel

**DOI:** 10.1016/j.geoforum.2018.10.007

**Published:** 2020-03

**Authors:** Nina Garthwaite, Mick Hatter, Jon Jonn, Stewart Maxwell, Frank Benson, Lynne Friedli

**Affiliations:** a 33 Morris House, Roman Road, London E2 0HP, UK; b c/o 33 Morris House Roman Road, London E2 0HP, UK; c 22 Mayton Street, London N7 6QR, UK

**Keywords:** Austerity, Homelessness, Benefit claimants, Poverty, Testimony, Work

## Abstract

An edited transcript from a conversation between Nina Garthwaite, a former receptionist at a homeless hostel in London and four of the hostel residents: Mick Hatter, Jon Jonn, Stewart Maxwell and Frank Benson. In it, they reflect on a series of discussions held in 2015 and 2016 chaired by Dr. Lynne Friedli, a freelance researcher with a special interest in mental health and social justice. These discussions covered topics from welfare benefits, housing, politics, work, rest and everything in between. Reflecting on these discussions, the men critique the institutional structures that frame their experience of homelessness and joblessness. They argue that these institutions are failing the individuals that they claim to serve. They also reflect on their encounters with academia through this project. They question whether academic research adequately engages with those experiencing austerity first hand. Finally, they question whether it is sufficient to research and discuss these issues without a corresponding focus on action. The paper includes commentaries by Lynne Friedli and Nina Garthwaite, reflecting on the discussion and the use of personal stories, as well as the complex ethical issues involved in bringing together academic and non-academic voices. These include the impact of structural inequalities, power relations, the intersection of class and gender and the power dynamics that flow from the radically different positions of the authors and participants in relation to homelessness and employment and precarity.

*Stories are extremely powerful and have the potential to bring us together, to shed light on the injustice committed against us and they lead us to understand that not one of us is alone in this world. But our stories are also a commodity—they help others sell their products, their programs, their services—and sometimes they mine our stories for the details that serve their interests best – and in doing so present us as less than whole.*

Becky McFarlane, *Recovering Our Stories* event, June 2011 cited in Costa et al. (2012) p85

## 1 Introduction

**Nina Garthwaite**

In 2010 I took a job as a part-time receptionist at a homeless hostel in East London. At the same time I was setting up a company, In The Dark, which specialised in audio storytelling. The reception job was intended to be a short term thing. I ended up staying for 6 years.

I saw both parts of my working life, audio and reception, as being engaged in a similar activity – “listening” or perhaps “receiving”. But in both cases this was not a passive affair. Just as in audio I enjoy talking and thinking critically about the power of the media, as a receptionist, I couldn’t help but engage with residents who wanted to talk and think critically about their experiences of homelessness, benefits and hostel life. It was because of some of those conversations that in 2013 I offered a nine month workshop in the hostel to teach the basic skills of audio storytelling, which two of my co-authors here participated in. The aim was to show how stories are used by the media and possibly how to take power of your own stories. They learned to use the recorder and made short pieces about each other, which were then played to other residents in an evening of collective listening. For me, one highlight of this project was sitting at reception one day and seeing a resident doorstep a slightly worried but otherwise game member of hostel staff. “You’re not doing an expose on us are you?” he (half) joked.

In 2015 and 2016 I was invited to be part of a 2 year collaborative residency, “Hubbub”, that explored “Rest and its opposites” through interdisciplinary research. Here, I met Lynne Friedli. The initial impetus for our collaboration was to share the findings from Lynne's research on workfare (work for your benefits schemes)^1^ with hostel residents, many of whom have extensive experience of unemployment and the benefits system. We decided to run it as a discussion, so that the residents would have a chance to share and analyse their experiences in an academic context.

The popularity of the first discussion led to a series of four conversations over afternoon tea with residents, with topics drawn from Lynne's research: 1. Work for your benefits: is that fair?; 2. What's wrong with work?; 3. Is rest possible? and 4. What’s wrong with the system? The conversations that took place deepened into ongoing dialogue, collaboration and involvement of the residents in Hubbub events.^2^ At the end of the project four of the residents joined Lynne and I to present at the Royal Geographical Society’s Annual International Conference, and agreed to co-author an academic paper on our experiences.

For me, the power of this project was in its potential disruption of the power dynamics within the homeless system described by the residents. A little like the microphone, the collaboration with academia challenged who was entitled to describe and critique their experiences. But looking back, I wasn’t sure if “disruption of power” was just a flattering way to think of the project, that perhaps served my ego more than anything else. When so much of what was discussed, and what you will encounter here, was about the powerlessness hostel residents and benefit claimants feel, I was interested to know whether this bringing together of academic discourse and corridor conversations was really a “zone of resistance” or that this was simply my feel-good interpretation. Nearly a year after the project ended, I (N) went back to find out what this experience had meant to those that had taken part. We agreed that a transcription of this encounter was a way we could co-author a paper.

I spoke to four of the participants, Mick Hatter (M), Jon Jonn (J) Stewart Maxwell (S) and Frank Benson (F). By this time, my role as a receptionist was now formally over. While I (and the residents when I discussed it with them) do not think this ever made much difference, any potential power drawn from that role was now removed. We sat in the garden of the hostel, a place we had met and talked many times before. The atmosphere was jovial, we were pleased to be all together again, they were relaxed and uninhibited. I put my recorder on the table – the same one that had been used for the workshops. They said they weren’t bothered by it. I didn’t have a list of questions, but planned to ask about how the different aspects of the activity had stayed with them, from the issues discussed to the interactions with academia.

Two of the men, Frank and Stewart, are no longer living at the hostel. Frank is now renting privately, while Stewart’s accommodation, while still considered “sheltered accommodation”, provides him with his own flat. Jon and Mick still live in the hostel, where they have been now for 7 years.

The hostel accommodation consists of a single room. Most have an en-suite shower room. They have no means of cooking their own meals, though there is a subsidised canteen in the building. They are not allowed guests in their rooms at any time. They can socialise in communal spaces on the ground floor, which are shared with around 170 other residents. They are not permitted to bring alcohol into the building. The rent for this is approximately £960 a month. However, provided that they are eligible for Housing Benefit, the council directly pays the hostel this amount. Residents also pay a Service Charge. This varies but at the time of the discussions most participants paid approximately £16 a week. This includes, amongst other things, utilities, daily cleaning of their rooms, and breakfast.

Most of the men are on benefits. Many are on Jobseekers Allowance. If the residents are deemed to have broken any of the conditions of their benefits the Jobcentre will stop their benefit payments for between a month and 3 years. Arriving late for an appointment, for instance, could be enough to get their benefits stopped for a month and there are many other possible causes. In such cases, the residents are left with nothing to live on and may also lose their housing benefit.^3^

Below is an edited transcript of the conversation I had with Frank, Stewart, Mick and Jon reflecting on the project. It has been edited to reduce the length. This was mainly achieved by removing repetitions and diversions and I worked to maintain the structure, flow and content of the original conversation in editing it down. A draft of this transcript was shown to my co-authors. Three made minor alterations to their own words to aid clarity (rather than to change meaning). They all signed off on the final transcript and summaries.

## 2 The Discussion

1. *The conversation began by reflecting on what they had taken from the discussion sessions on work, benefits and people's experience of the system.*

N: It’s almost a year since we finished the project. What has stuck with you?

F: I think for me it was the system of working for your benefits. The Jobcentre makes us work for free for others for our benefits. So even though we are getting Jobseekers Allowance they say “if you want the benefits you have to volunteer”. They call it “work programme” or “training”. I did this with the London Credit Union. I was getting the same benefits but the Credit Union weren’t paying me anything, so when it came to my actual pay per hour, I was earning £1 an hour with them. It was sold with different names and titles to it to make it sexy, but at the end of the day I was working for somebody for £1 an hour. It is horrible. And I just read in the paper today that the government want to abolish this because now they realise it’s just taking advantage of people.

M: On Friday I got a call from a company, a warehouse, and the guy said to me “are you working at the moment?” I said “no I’m not”. He asked “Are you on job-seekers allowance?” I said “yes I am” and he said “Oh that’s good!”. I said “why is that good?” he said “because we do a four day introductory to get a qualification to work for this company”. I said “now hang on a minute, I’ve got two qualifications as a Warehouse Assistant, I’ve done a two week course of manual handling and health and safety, and I’ve also got a CSCS [Construction Skills Certification Scheme] card. Now, if that’s not equivalent to what you want for working in the warehouse, let me think about the job”. It’s because the company will be getting paid to train you. Why can’t they just employ you? If you’re any good take you on, if you’re not, you’re off.

J: It’s tactics.

M: Everything’s messed up with zero hours and part time work. The amount of part time work I get sent on my phone is unbelievable.

F: But at the end of the day, what you’re going to be making is less than what you get if you’re not working.

M: I’d be exactly where I am, I might even be losing a couple of quid.

J: There’s a great marriage between the government and the companies. You’ve got lots of people that have set up companies making money out of people who haven’t got jobs. They get paid subsidies by the government, so they’re making money, but you are just jumping from one course to the next course. They just move you around. And they’re doing that with people who are seeking employment.

2. *Jon is not currently on benefits and is running two small internet businesses. For him and for Stewart the problems go beyond the particulars of finding employment through the Jobcentre. They question the assumed models of work that the benefits system seems to serve and, by extension, the framing of the discussions.*

J: Now, similarly, for people like me that are setting up businesses, they want to tie you into loans or grants. I actually remember the benefit people asked me if I wanted to start my own business, and I realised that the Jobcentre asked a lot of people that. Luckily I had that intention anyway, but a lot of people I know said “yeah” but they knew nothing about starting your own business. It’s just a way to get them off benefits…

F: …they just sign them off…

J: …and they’re not entitled to anything. And then it goes off the books so they can go back to parliament and say “this week this many people went back to work”.

It’s a big game. There are not a lot of jobs out there. They like you to think there are jobs out there. There’s not a lot of jobs out there. Then somehow you’re tied in and if you have a loan in a business that’s not doing well, you’ve got money to pay, the same thing that they’re doing with students as well. Tie you with loans.

One problem that I had with the discussions with Lynne is that this work thing that we discussed initially was heavily based on someone going out to work, being employed by someone else…

M: …and getting nothing…

J: …and obviously that conversation comes down to you not getting a lot from working for someone else, either financially or whatever else. We didn’t really discuss the broader types of work. I could work for you or we could collaborate, or I could work for myself. That’s work as well so what else might I gain from that? I think the questions we were discussing were heavily about going out and seeking work from others which for me, the way the world is today, it’s really not relevant. There’s a lot of diversity of what we call work nowadays.

S: What is work and why is it changing? And why do we have this attitude that we don’t want to go into a 9-5 job and work for a boss? We’re not going to be bossed around. We’re not going to work a 9-5 to get a wage, have a little bit of food, have a drink at the weekend, retire having made money for a boss. It’s like you’re a slave. I’ve said this before, it’s a form of slavery…

J: …it *is* a form of slavery…

S: … and you have that sense that you could be more valuable by doing something different, by doing it another way.

J: Exactly. But did the conversations give us that choice? This was about work, as an employment, that’s how it came across to me. That’s not the only type of work…

M: that’s working to live isn’t it?

S: I think what we’re all feeling really that we need to have the sense of freedom, and a sense of control, and a sense of being valued. Mick’s just feeling that he’s being used, and people are using him because they’re making money out of him and he’s not getting anything out of it. And we’re being used, we’re being told to “just be quiet, don’t say anything, don’t complain about the system, we don’t want to know really, we’ve just got these rules, you come in, do the work, we get paid, you get nothing, and everybody’s happy”.

M: Except the bloke that’s going to work

F: Exactly.

N: Jon, you mentioned that you felt we didn’t have a wider enough discussion about work. Was that because of the focus of Lynne’s research at the beginning was the Jobcentre and the benefit system?

J: Now, it’s funny that you say that because I was claiming benefits as well, what I had was called “Working Tax Credits”. But for some reason they stopped it. Some of us wanted to avoid being put in a box by the Jobcenter and being told what to do. We were finding other ways to work, such as running a business, that gave us more freedom. But they shifted us away from Working Tax Credit – they’ve stopped mine. This is what was interesting to me – they wanted to shift people away from Working Tax Credits and put the emphasis on employment. So this “work” thing that we’re talking about, I think Lynne needs to spread it a bit wider, and then see a clearer picture of what’s going on out there. That’s what I was slightly disappointed about in the discussions. But I liked the energy you know, at least talking about it so that residents here know what’s going on.

N: See my personal feeling is that Lynne brought a question about working for your benefits, but it moved on and created a forum where you could talk about alternative views of work and different ideas of what good work could be like.

S: Yes we did talk about that because Jon’s always wanted to start his own business, Mick’s always looking at jobs and working for someone else, Frank’s always looking for opportunities but ended up working for free for a bank. I have an aspect, Mick has an aspect, Jon has his aspect and Lynne’s bouncing between all the ideas.

J: But we didn’t get to the core of what the problem was. Work is a broad subject. What’s work?

S: Yeah. The topic was mainly around what is the meaning of work and Lynne brought that academic side to it and brought everyone’s conversations to a meaningful conclusion, but it made me think: what is the point of work? Or is there a way of being more valuable in the world, which is what we should be really. Well, this is my point of view.

3. *All the men agreed that the discussions helped them feel less alone with their experiences and more inspired to take action.*

N: So do you think there was a value in having these discussions?

F: Basically it raised questions we were not used to discussing before so it was a change for us, I felt.

J: We’d thought about it but we’d not really discussed it…

F: …out in the open…

J: …I must admit though, it’s something that you must have noticed that me and Patrick [another resident] would talk about in the corridor in the morning, I don’t know how often you saw us, and people would say “what are you lot talking about? Are you trying to start a revolution or something?” and we would laugh about it. But it was interesting to engage in the same conversations with other people around as well, so for me, I took a lot out of it with regards to seeing others’ point of view as compared to what I’ve always thought about it.

M: It brought out that everyone is an individual, everyone has different needs. I mean, in here, it’s unbelievable, you’ve got people taking drugs, you’ve got people on the beer, you’ve got people walking about shouting to themselves.

S: It made me think that I want to do something…

J: …which is a good thing…

S: …it’s alright talking, but I’ve got to do something.

J: Yeah but it starts with talking isn’t it?

S: Well, I suppose it does. What makes you take action?

F: I think just for each one to articulate what’s inside out and find out that others share the same thing, it’s a very big thing.

S: It makes you feel part of something, it’s that feeling of being part of something.

F: it’s not like you’re the only one with this problem

4. *All of the men felt that there were barriers to talking openly and critically about their experiences with the benefit system and about work in general. Some were worried that it would have direct consequences on their benefits or housing if they were heard talking critically about work or the system. All of the men felt that the employed have assumptions about the unemployed and that these assumptions prevent the men’s criticisms from being taken at face value.*

S: This is 2017, it’s the 21st Century and you can’t have a discussion about what is work and how we should be more productive, because if someone’s listening to you they’ll nip down the Jobcentre and say “he doesn’t want to work, maybe you should cut his benefits” do you know what I mean? Why should you not be allowed to have that conversation? There’s still that feeling – you still have to be doing this and be seen to be doing that, working within their methodology.

N: Mick, I remember at the beginning you didn’t want to come to the discussions initially because…

M: …I thought we’d all be targeted. We’d start making trouble…

N: … in particular you didn’t want to talk about how someone might not always want to take work that is available to them because it isn’t worth their time…

M: …well that’s quite true…

N: …and that’s something that you were uncomfortable talking about openly in the beginning.

M: This place [the hostel] is a catch 22. Unless you can get a good wage you can’t afford to live out in a flat, you’re fucked. Aren’t you? For argument's sake – if you say about zero hour contracts, you might get one day a week, two days, four hours, where are you going to pay your gas bill? You’re still going to have to go back on dole money, in other words, you’re stuck here.

N: Do you think the feeling that it’s a taboo to say this, is it based on experience? is it a feeling that you shouldn’t talk about it, or is there an actual risk to discussing these questions?

J: For me it wasn’t a taboo, but for me I can see if you don’t get up at 9 to go to work, people look at you differently. Therefore that’s a problem. People don’t think that you could be working in some way even if you are not jumping on a bus to go work for somebody else.

N: so do you think that people make assumptions about people who are unemployed?

J: Well I did. You do. When I was working for someone, if you didn’t have a job, I looked at you differently. Why haven’t you got a job? If you’re disabled that’s different, but if you’re an able-bodied man, or woman, why can’t you work? So you do get judged. That’s not new.

The problem is, if you’ve got your own life to live you don’t look at someone else and want to understand why they’re not working. Mick said he wants to work, but if you check what he’s going to earn, and how he’s going to live, there’s a problem. For instance a nurse told me she goes to food banks. My jaw dropped! £1300 a month, pays her rent, she’s married, luckily her husband earns money as well, but she still has to go to a food bank to feed herself. And I’m like: what? That nurse is someone who is actually working and she can’t afford to live. That took it to a different level about what work is all about.

S: I know. There’s a housing situation that’s desperate. There’s a work situation where you can get work if you want to but it doesn’t pay your housing; it doesn’t pay your housing, doesn’t pay your food, doesn’t pay your bills, it’s not enough to give you a life.

M: Even people like old age pensioners are using food banks.

J: So this is not just about people being lazy anymore, this work thing, it’s not about that. If you’re working and you’re still going to a food bank, there’s a problem. Kitchen staff here have told me “why don’t you go out and get a job?” and I look at that person and I think “what? I probably earn more than you do, because you see me here that doesn’t mean I don’t work. You see me reading the paper in the corridor in the morning, don’t assume I don’t work” but that’s the mentality.

S: that’s the lack of understanding

5. *All the men felt that Lynne’s presence as an outsider and researcher enabled them to express ideas they wouldn’t otherwise, and felt that the ability to share ideas was very valuable.*

N: What difference do you think it made for Lynne to come in?

J: It added a weight to what we thought about, and for her to research it confirmed to me that what we’d talked about privately was important.

F: In general people don’t like to talk and listen to others. I mean in general, if you go anywhere and you want to talk to someone, people just don’t want to talk…

S: …I think there was a structure to it, you had to meet at a certain time, you had to have a certain amount of discipline, you had to talk and listen and there was a structure to it, and it was interesting….

F: …and at least the fact that you knew when you said something others were listening. I mean I notice when I go around London, you know central London, West End, it looks like a crowd, but actually people are all isolated. You can’t talk to ‘anybody. First of all, they’d ask “why are you talking to us?”. We are living in isolation but it looks like a big crowd. It’s very interesting.

J: Why do you think Facebook is so popular? It’s the same thing.

F: Because people want the impression that they have 5000 friends.

J: No, it’s not just that…

S: …look at my lamborghini…

J: …no it’s not just that. People go on Facebook because it’s an outlet, they can release whatever they have got to say, so it doesn’t matter if someone is listening or not…

F: …at least they’ve said it…

J: …they’ve said it, they’ve got it out…

F: …it’s recorded somewhere.

J: …they’ve got it out somewhere.

F: If you say it by yourself in the street, they’ll think you’re crazy.

J: Exactly. So people can engage, which is very important to their wellbeing.

M: Yeah you couldn’t actually talk as we were talking in front of staff. You could talk to Lynne because she’s an outsider and probably done research in other areas.

6. *For the final discussion session Nina made a particular point of asking staff, including the senior management, to attend. The theme was “what’s wrong with the system?” and she hoped something positive would come from a dialogue between staff and residents about the wider context in which they both were operating.*
*Significantly fewer men turned up to this discussion, and of those discussing here, only Stewart and Frank were present. They felt that the ambition of dialogue was not achieved in this session and that the conversation was shaped not by the wider systemic questions posed but by hostel’s defence of their own role in the resident’s lives.*

N: Well some staff did drop in and in the end we did make a point of inviting staff – the discussion “What’s wrong with the system”. Far fewer people turned up to that one but for those who did, how do you think that went?

S: Well it felt a bit of an “us and them”.

N: I was hoping is that there would open up a dialogue.

F: It didn’t.

S: But you see that’s the problem, there isn’t that kind of respect and exchange to say “what do you want? You’re a grown man, you’re not a seven year old”. There was no open mind and “oh I didn’t realise you were thinking like that”. But I wish these discussions had have evolved, the first one was always going to be awkward.

F: I think there was a comparison between us and them, between the residents and the staff because they were working and had jobs and we didn’t. So I felt somehow that they were looking at us as jobless.

J: But isn’t that a reflection of society?

F: In a sense I felt that they were saying “we have jobs, you do not, and we have to manage all your benefit headaches and housing”

S: “Just do as you're told.”

M: It’s like the Jobcentre.

N: How was the nature of the conversation with staff different from those that were mainly residents?

S: I think it was the tension, they were careful about their responses, it wasn’t friendly, there was no banter, you had to be careful about what you said.

N: What do you think the source of that tension was?

S: It was that the MD was there. His position was “you come here, we give you somewhere with a shower”.

J: So does that mean you have what you need to build a life? If the Jobcentre find you are not working, they say “get them a course to do, get them on the ladder” but after … I know a guy here who’s got 21 certificates. What’s that about? 21 certificates. He goes to courses, he’s very bright, yet he comes out with no job to go to. But they say “we’re giving them courses to do and they've got a job waiting.” Jobcentre says it, the hostel follows the same line.

N: So do you think it was useful to have that conversation with staff?

S: I think it was interesting, yeah, but I think it’s a case of whether people are willing to move forward and work together and to say “I didn’t realise you were thinking *that.* Maybe we should be doing *this*”

7. *The men felt that in general the hostel’s actions are defined by financial concerns and that this was detrimental to the needs of residents.*

N: Do you think that in a hostel like this should engage more in conversations about the social and political environment that residents are operating within?

J: You’re talking about engaging in the wider social questions, but are they even giving us the practical help we need?

S: I had that stroke just as I was moving out, and I asked for help, I asked for a van, and I got nothing. No one phoned me to see if I was ok, what happened… I mean, nothing, no one said “can I give you a lift to a hospital.” nothing.

J: Listen, this is a business. It’s a charity, but it operates like a business. That’s why they don’t like people giving food away.

F: Because they want to sell the food here.

J: Thank you, they want to make money.

F: I worked in a restaurant for three days, you know, a kitchen porter, and the restaurant, you know it was very posh, they gave me cake, beautiful cake, and I brought it here, the management came saying “don’t bring food and give to people!”. I said “come on I’m not giving to dogs, I’m giving to my friends” “no you can’t bring it in.”

S: It's awful isn’t it. It’s really awful.

F: I was sat at reception giving the cakes away they came like doberman dogs, “no don’t do it”

S: I just don’t get it.

M: Can I say something, now, since like probably last Wednesday or so, you’ve got either two or three Tesco vans coming here round about 9o’clock or 10o’clock at night bringing boxes of bananas and different types of fruit and everything. Now we have to pay for our breakfast as part of our service charge. They get free bread in the morning. If you go and ask them for two slices of toast it costs you. So how does that work out with your…

N: …so they’re selling…

S: …they’ve always done that with donated food. Sometimes they’ve given that out and sometimes they’ve sold it.

F: For tax purposes this is a charity, that’s what it seems like.

S: Solely for tax purposes.

8. *Residents felt that they were trapped in housing that was inadequate but inescapable and that this made them powerless. They felt that this lack of control residents felt over their lives was likely to have repercussions for their mental health.*

J: You know what happens when someone is trouble, I’ve seen this not once not twice, they will tell you “I will pay for your deposit, move out”. Why do they tell you that? They know the deposit is nothing, it’s worth the trouble to get rid of you.

N: I’ve heard staff tell residents who have got angry “there’s the door, if you don’t like it here you can leave.”

J: Where they’re coming from, they think it is the best place – So you’ve got to understand the mentality of people who work here. They assume when you walk through that corridor and its painted and it’s nice, you should be happy with it, that you should feel lucky to have a roof.

M: Because someone else is going to jump in.

J: Because someone will come and easily pay and keep his mouth shut. But you go out there and you’re not earning enough to live in a proper flat. Next you know, you’re looking for the next hostel.

For me, the reason I’m happy here, is it’s clean. I couldn’t live in [another local hostel] else I’ll end up killing someone or someone will kill me. That’s the difference. And they know that.

M: Yeah, I was told that they’re even allowed to bring their cats and dogs in there.

S: But if it’s so nice here, why are we complaining?

N: Well it’s nice compared to other hostels, but that doesn’t mean it’s adequate housing.

S: Well yeah, should people be living like this? No.

J: And it reflects society…

S: …you should be in your own place…

J: …freedom, peace of mind.

S: You should be able to support yourself. I’ve got a door key now. Nobody tells me what to do, I can have a drink in there.

J: Can you imagine that freedom and what you used to live with here? It’s a big thing. People out there…

S: …I can do whatever I please!…

J: … they’ve got mental illness, right? They’ve got mental illness, you know why people are suffering now today? It’s no big thing, it’s these little things…

S: …you’re being controlled….

J: …yeah man, no one wants to be controlled, but you don’t have a choice…

S: …no, you don’t have a choice…

J: …so it comes back to work…

S: …you have to do this…

J: …they’re telling you you have to work zero hour contracts, of course you have to eat so you’re going to work…

S: …you have to eat, yeah…

J: …but guess what? Slowly but surely it’s affecting you. Mentally…

S: …it eats away at you…

J: …you’re working for someone and they can tell you whatever shit they want…

S: …well that’s another situation…

N: …and then it doesn’t necessarily pay…

F: …it’s worse than getting no work…

M: …it’s just going round in circles because let’s face it, if you ain’t earning enough money and you aren’t even in a flat, you can’t pay the electric bills, yeah, you can’t pay the gas, you can’t pay the bills…

J: …you’re stressed out mate…

M: …if you’ve got to use a food bank, it means there’s something wrong.

J: Frank’ll tell you. He wants to come back.

S: Ah no, no way.

M: you want to come back here?

F: yeah of course man.

M & S: you need to get a life [laughs]

N: Frank, you’ve now left the hostel and you’ve found somewhere to live – why is it you would want to think about coming back?

F: I think I’ve always… I’ve lived here 6 years, there are pluses and minuses…

M: …yeah I’m the same…

F: …the pluses is that you are well taken care of in that your room is clean, you come down your food is ready, go to the lounge, so it’s almost like a community. The minus is that the management was treating us like dirt, like kids, so this is what was really the tipping point for me.

9. *The residents all felt that it was risky criticising the hostel because of their vulnerability. They saw similarities between the precarity of their position in the hostel and in the workplace for the same reason.*

N: Do you feel free to talk about these things openly in the hostel?

J: They’ll make your life hell. It may not be tomorrow or next month, they will come for you.

F: They will make your life hell.

S: You’re incredibly vulnerable.

F: You could be on the street in no time.

S: You could be on the streets with a week’s notice.

N: But we’re just talking about freedom of speech, expressing ideas.

S: Yeah but it doesn’t exist to the extent that it should do.

J: Yeah of course, they don’t see you like that, they see you as a threat.

F: Even in the example of the company I worked for, we had a meeting, all the branch managers and the head said “we want everybody to be open and” – what do you call it – whistleblowers? “We have a policy to protect whistleblowers” and I sent her a direct email about serious trouble in the branch. I was the first one they let go. What policy to protect whistleblowers? All these things that they tell you about policy is on paper. The reality is different.

S: The thing is – what rights do you have?

F: They tell you “we have a policy on whistleblowers if there is a problem, forget about the branch manager, tell us so we can correct it” the minute you tell them you find out no they are in bed with the branch management, fuck you, get lost. You know, from the experience of so many jobs I talk here. What they have on paper is different from reality.

S: Well I’m not sure it is actually because you do have rights.

F: Well I’d have to go to court and I can’t afford to go there because I couldn’t pay the £400.

S: Well exactly, where are you going to get the money to pay?

J: That’s why I keep saying to you guys, whatever is happening here reflects society. This system they’ve got here, let’s say you’re looking for housing, they won’t help you. If you go to the council and ask them why not, they’ll say where you live, your contract agreement says it is a home, so you are not a priority. Then you have to ask yourself, why is that? You’re not allowed to bring anyone here, you live alone, you can’t cook, how is that housing?

F: You can’t drink…

J: …exactly. You mentioned the food as well, you can’t give away food. So it’s not housing they’re not helping you out are they?

F: They’re just covering it as a form of housing.

J: And yet because they are a charity they get donations and people see it as helping you out.

F: of course

J: Isn’t that is what is happening in society today? We are looking after the country, but realistically, that’s not what they are doing. They are looking after certain people, the rest of you, go fuck yourself.

S: Yeah but the thing is, you have to get yourself out of here.

J: No but forget about me, it’s not about me.

S: You have to get yourself out of here. Mick has to get himself out of here. I have to move myself on from sheltered accommodation. I have to do it. Whether you can get the help from them, which you don’t. How do you get out of here?

J: Without the rules or the arrangements with the council to help you do that, you can’t get yourself out unless you’ve got the money and can afford to, and for me, that’s where the problem lies. This gentleman here, Frank, I’m sorry to hear him say that he wants to come back…

S: …Christ that’s unreal…

J: …how many people since I’ve been here, have gone out there, and then come back here.

M: Because they can’t face the world.

J: Not that they can’t face the world.

S: I think it’s because they are struggling, it’s the money.

J: The money situation, they struggle. See, people need to understand, they say “yeah I’m moving, I’m happy about it” but this is what people don’t take into consideration when they move out: whatever job you’ve got, right now if you want to pay bills you’ve got to pay a certain amount or you’ve got to live with people to be comfortable. How many people have gone out and wanted to come back here? You can't afford to live out there, even if it’s a room. And therefore it’s difficult.

Now the hostel should be working with the council to say “now listen, we’ve got boys here living in this hostel for so and so amount. If there’s social housing, perhaps let them be a priority”. That’s what other hostels do. You go there, you fill out the form in a certain way so that you are a priority. We’ve known people who have lived here for years and they have gone to [another local hostel] and within three months they’ve got a flat. What does that say to you?

S: Perhaps more interaction with the management. More meetings with the management. You’ve got to have that – that’s got to happen. There’s got to be a realisation that they’ve got to do that or that that conversation’s got to go on.

J: Let me ask you a question, why do you need the management at all?

S: Well that’s a good question, you probably don’t. Because you have all the solutions.

J: Not necessarily. But have they got the solutions for us?

10. *The residents felt that though the discussions were important, it was a serious shortcoming that they did not result in any specific actions*

N: Ok so can I ask, having the discussions, it didn’t really make a difference to these issues – or did it?

F: It just made us aware of stuff.

J: I think it made residents aware.

S: The thing is people have to take action for themselves they have to decide that they are going to take action, they are going to change it.

J: That’s where my problem lies. We’re having all this discussion, where is it going? If you remember I did ask this question earlier, where is this going to? If we can have a discussion and it affects something, I’m happy to be a part of it, but if we’re just sitting here talking, it might be good for the mind…

M: …medicinal purpose…

J: …it might be good for the mind but what is it actually achieving? That’s why I tried to ask Lynne, where are we going with this?

M: What’s it leading up to?

11. *At the end of the project the men presented a paper, alongside Lynne and Nina, at the RGS Annual International Conference. Although they all enjoyed the experience, they were unanimously critical of the academics that presented before them. In particular, they felt that the feminist perspective these particular researchers were speaking from represented a distortion of the issue of austerity, something they felt academia (and society in general) was increasingly guilty of. However, they strongly felt that academics have a role to play in affecting positive change. For this to begin the men felt that academics should listen to people who have first hand experience, like them.*

N: Can I ask then what were your impressions of presenting at the Geography conference?

J: Actually I was really upset when I came out of that place.

S: Oh were you? Why? I really enjoyed that, I thought that was absolutely brilliant.

J: We walked into a place where – and I enjoyed it because of this – we walked into a place where two women were talking about austerity, but they were talking about something completely different from why we came in there. Now this lady started well, the one from I think Leicester, she was talking about women suffering abuse in the home, I think that’s how she started. In the family. And she started well and I was quite interested in what she was saying, and I thought “ok, so the dynamics of family are important” and then all of a sudden she shifted from that and started bashing men. And that reflects the academic society as we’ve got today. We’ve got two women that picked one subject and another subject and merged them together and found a link, and let me tell you what it is. Those two women are feminists, they came in just to bash men. Straight up, and you know what, I see that on social media all day long, but I didn’t take them seriously, but that particular day, that’s when I realised how serious our situation is.

M: It’s like they hated men.

J: She didn’t even speak to women that were abused, she spoke to people who look after women who are abused. So you are drawing a conclusion that men are the cause of the problem. And this is an academic, what she says is important, she’s drawing a conclusion that men are the problem.

S: It’s not that simple is it?

J: Now I had a look around. There were about 60% men, 40% women in the room. When they were talking, I was listening to them, these were academia sort of people, when they finished talking and they asked questions like “anyone got anything to say?” no one of them said anything. It took me and Steve, right? To say “we disagree with what you are saying. We can relate to the same problems you are describing, so what’s that got to do with you bashing men?” One of them actually shrugged her shoulders. She didn’t care – she had her agenda and she came and said it. And this is the interesting bit: when I was saying what I was saying of “listen I can relate to what you're talking about, it’s not only women” half of the room were nodding their heads. Even Lynne afterwards said “yeah he’s right”. So that’s what we’re dealing with here.

Now, I don’t know if it’s a deliberate way of shifting the agenda or conversation away from what is actually happening. They’re mixing austerity with other things and therefore confusing the situation, because no one actually wants to deal with it. Like those two women were doing. They didn’t come there to talk about austerity, how it affects family, which is how one of them started, or abuse in the family situation. They just accused men. Three guys came to talk to me afterwards and one of them had a tear in his eye, no one saw it, because he said “what you’re saying is absolutely right” his dad went through the same thing. So we’ve got…

F: …there’s a huge movement now, a huge huge movement against men…

N: …see I don’t think personally those women were there to man bash, but maybe they’ve got into a prism of looking through the world which is maybe a feminist prism, and that yeah it makes them blind then sometimes to…

J: look…

N: …but you know that’s also how academia works as well…

J: …that’s how it works!…

N: … which is different people have different prisms through which they look at the world. I think it’s interesting to look at something through a particular prism and say “ok let’s look at this through a feminist prism and what does it look like, let’s look at this through Marxist prism, what does it look like, let’s look at this through a libertarian prism and what does it look like” but then you also need to step back or make sure there is a dialogue between them…

J: …thank you, and that never happens and those people talking, they look down on what you, as a non-academic, have to contribute to the conversation

S: Well, I mean that’s a problem there, if that exists, and it shouldn’t exist, because people should be saying “we’ve got a system that’s inclusive! It’s not feminist, it’s not marxist, it’s not social elites” our system is inclusive, it doesn’t have an agenda, so what is it about valuing another human being? Is it as simple as that?

J: exactly, you are right!

S: Listen, you’ve got an opinion, ok? Tell me what it is. I’ve got an opinion, tell me what that is. How do we go forward?

J: Yeah but that’s not what happens is it? We look at it from prisms.

S: So why doesn’t that happen?

N: But I think it’s ok for them to look at it through prisms, as long as….

S: …but there’s a blindness isn’t there?

J: Thank you!

S: There’s a blindness. They don’t even know that they don’t know. They don’t even know that they’re doing it.

J: you could argue they look at it in prisms because it helps move forward quicker, or whatever, but like Stewart just said, there might be a blindness that something that is very important to the conversation.

S: That’s right! This guy has got an idea that’s going to change the world! And I’m not interested.

M: Leave me out!

N: So would you say that this kind of collaboration/working together could be helpful to open up a more equal dialogue with academics?

J: Absolutely

S: Yes we do need it yeah.

J: Absolutely!

N: So in a way this links back to the project – Jon you mentioned the research that had mainly happened through speaking to women’s caseworkers rather than the women themselves. Do you think there’s a potential problem with academics, if you think about the people who run hostels like this, people who run various homeless charities, do you think there’s a problem where those people become gatekeepers to the voices of the people who are actually going through it?

J: You see, you just said it. They become gatekeepers. And the people that write policy or politicians talk to these people, and they don’t talk to the homeless people actually that are going through it, and these people, why do they talk to them? Let’s face it, some of them get finance for what they do and therefore whatever they write has to match certain positions for them to get the money. Yeah they might touch on the situation is, but let’s not beat around the bush, they’ve got to write certain things for them to get the finance, and therefore it doesn’t actually fit what is happening on the ground. There’s a lot of that happening.

M: A lot of covering up.

J: A lot of covering up. Now these politicians are very clever as well. They’re not going to go to someone like Lynne who is actually going to tell them what is going on, they’ll go to someone else…

F: …who’ll tell them what they want to hear…

J: …to tell them what they want to hear. And then guess what happens, they’ve got the media on their side.

F: Of course

J: Investigative journalism is out of the window nowadays. So the people who have been through this situation are not being heard, at all. So if I’m watching TV and a politician is talking, I just think “you’ve no idea what you are talking about mate”.

N: So if we were to do the project again, what would you change about the project?

J: I don't just want a verbal exercise, I do enough of that as it is.

S: Talk is cheap.

J: But if it helps to start a conversation where the people who have the power talk to people like us, people who are actually on the ground, that’s my job done. That’s all I want. Because if you are talking to people in a chain, agencies, charities – people who have to talk to other people – you’re not getting the real story, and unfortunately that’s what the problem is.

N: And do you think academics have a role to play?

J: Of course.

S: Absolutely, absolutely yes.

J: Of course they do. And if they are not talking to the real people they are just…

M: …they're making it up, aren't’ they.

S: They’re making it up, yeah.

Below is a short commentary from Lynne on this discussion.

## 3 Encountering austerity, encountering academics: reflections on research and activism

Lynne Friedli

*J: 'Let's face it, some of them get finance for what they do and therefore whatever they write has to match certain positions for them to get the money*…*.*… *and therefore it doesn't actually fit what is happening on the ground.'*

The series of conversations described here started with a discussion about my research on workfare, (the requirement to carry out unpaid work in order to receive welfare benefits), and culminated in a paper, jointly written and presented with five residents at the Royal Geographical Society annual conference *'Encountering Austerity'*. What began as a debate on the benefits system*: 'work for your benefits – is that fair?'* developed into a deeper inquiry, in response to the request from residents for more opportunities for these kind of conversations and informed by residents' experiences of benefits, employment and homelessness.

The collaboration was not planned, which meant that the aims of the inquiry, the meaning of consent, our positionalities and potential benefits and risks for everyone involved, above all the hostel residents, were discussed and negotiated as the work progressed (Rose, 1997, Centre for Social Justice and Community Action, 2011). The troubled and troubling questions that surfaced as our encounters deepened concerned complex relationships of power and accountability and the ethical challenges of developing a research partnership under conditions of extreme inequality. As Stewart and Jon discuss here, what did being involved in this research achieve for the men who participated?

*S “* …*it’s alright talking, but I’ve got to do something*…*.*…*”*
*J “*…*it might be good for the mind but what is it actually achieving? That’s why I tried to ask Lynne, where are we going with this?”*

This question assumed a special significance because of how the men experienced the RGS conference. While our paper generated a positive debate, the men's attempts (in response to another paper in the session) to contribute views drawn from their lived experience as working class fathers were not engaged with^4^:

*J* …*“those people talking, they look down on what you, as a non-academic, have to contribute to the conversation”*
*J*…*.*…*“It took me and Steve, right? To say 'we can relate to the same problems you are describing, so what's that got to do with you bashing men .....when I was saying what I was saying, 'listen, I can relate to what you're talking about, it's not only women' half of the room were nodding their heads.”*

While this encounter was largely framed by the men in terms of *'a huge, huge movement against men'* (F) it was also seen as part of a broader failure to address the impact of austerity – ‘*what is actually happening’:*

*J: 'Now I don't know if it's a deliberate way of shifting the agenda or conversation away from what is actually happening. They're mixing austerity with other things and therefore confusing the situation because no one actually wants to deal with it. Like those two women were doing. they didn't come there to talk about austerity, how it affects family*…*.*…*'*

My work is concerned with the relentless labour of claiming unemployment benefits, at a time when the role of the 'job seeker' is more and more narrowly specified and supervised. In particular, my research for Hubbub focussed on the rise of psychological conditionality, which requires claimants to demonstrate beliefs and attitudes to work drawn from positive psychology.^5^ These developments, enforced through sanctions (the withdrawal of benefit payments), together with the precarious nature of the labour market, as well as the stigma attached to claiming benefits, make it increasingly difficult to express negative views about work, something hostel residents were acutely aware of.^6^

*S:* …*if someone's listening to you, they'll nip down the Jobcentre and say 'he doesn't want to work, maybe you should cut his benefits'*

In that sense, the debates at the hostel became spaces of resistance to mandatory positivity about employment, a passionate exercise in dismantling the grip of the work ethic **and** an opportunity to bear witness to an obvious truth: for many, work fails to deliver either emotional satisfaction or a wage you can live on. The **men’s** accounts of exploitation sit uneasily with the pronouncement of the Department for Work and Pensions: *“Just because you don't have a home doesn't mean you don't have to comply with your Job Seeker Claimant Commitment”.*^7^ Even more challenging, were the stories that exposed the contribution of the homeless industry to the high living costs/low wages equation that many hostel residents felt trapped by.

Drawing on these personal accounts for research purposes raises uncomfortable and unresolved questions about who owns people's stories, and the way in which when you take somebody's testimony, which is something that belongs to them, you are taking it and reframing it and you are using it. The 'donated labour' of being studied. *'Stealing the pain of others'* as Sherene Razack has called it (cited in Costa et al., 2012). This was exacerbated by the unplanned nature of our collaboration, which meant we often responded to opportunities (e.g. to present at RGS or to be part of Hubbub events) without in depth reflection on the differential costs and benefits for everyone involved (Centre for Social Justice and Community Action, 2011).

At the same time, the residents articulated a strong demand for the voices of homeless men to be heard:

*J: Yeah but it starts with talking isn’t it?*
*S: Well, I suppose it does. What makes you take action?*
*K: I think just for each one to articulate what’s inside out and find out that others share the same thing, it’s a very big thing. It added a weight to what we thought about, and for her to research it confirmed to me that what we’d talked about privately was important.*

The wider questions of how to shift mainstream research discourse on homelessness and the practices that flow from it, as well as the distinctive ethical challenges of the way we tried to work together (Dodson et al., 2007), remain unresolved.

## Footnotes

1 Friedli and Stearn (2015). ‘Positive Affect as Coercive Strategy’.

2 Further details of the collaboration in Friedli, L. and Garthwaite, N. (2017). 'Rest Denied, Rest Reclaimed' in F. Callard et al. (Eds.) The Restless Compendium: interdisciplinary investigations of rest and its opposites p173–182.

3 See report by the House of Commons Committee of Public Accounts on Benefit Sanctionshttps://publications.parliament.uk/pa/cm201617/cmselect/cmpubacc/775/775.pdf.

4 We hoped to explore these issues in a longer conversation, however, a response from the presenters of the paper in question was invited, but was declined. By contrast, following a similar presentation to academics at Hubbub, evident inequalities of class, status, payment and identity (e.g. who does and does not get to call themselves a poet and get paid for writing) were directly addressed and discussed by Friedli & Garthwaite in Callard et al. (2016).

5 Friedli and Stearn (2015).

6 South Wood Ivor (2011) Non-stop Inertia London Zero Books.

7 Statement from DWP at launch of Crisis Report Christina Beatty et al. (2015) Benefit Sanctions and Homelessness: a scoping report London: Crisis/Sheffield Hallam University Centre for Regional, Economic and Social Research.
